# Dose-Response of Superparamagnetic Iron Oxide Labeling on Mesenchymal Stem Cells Chondrogenic Differentiation: A Multi-Scale *In Vitro* Study

**DOI:** 10.1371/journal.pone.0098451

**Published:** 2014-05-30

**Authors:** Emilie Roeder, Christel Henrionnet, Jean Christophe Goebel, Nicolas Gambier, Olivier Beuf, Denis Grenier, Bailiang Chen, Pierre-André Vuissoz, Pierre Gillet, Astrid Pinzano

**Affiliations:** 1 Ingénierie Moléculaire et Physiopathologie Articulaire – Unité Mixte de Recherches 7365 Centre National de la Recherche Scientifique - Université de Lorraine, Vandoeuvre Lès Nancy, France; 2 Centre de Recherche en Acquisition et Traitement de l'Image pour la Santé, Centre National de la Recherche Scientifique 5220, Institut National de la Santé et de la Recherche Médicale U1044, Université de Lyon, Institut National des Sciences Appliquées de Lyon, Villeurbanne, France; 3 Imagerie Adaptative Diagnostique Interventionelle, Institut National de la Santé et de la Recherche Médicale U947, Vandoeuvre-Lès-Nancy, France; University of Rochester, United States of America

## Abstract

**Aim:**

The aim of this work was the development of successful cell therapy techniques for cartilage engineering. This will depend on the ability to monitor non-invasively transplanted cells, especially mesenchymal stem cells (MSCs) that are promising candidates to regenerate damaged tissues.

**Methods:**

MSCs were labeled with superparamagnetic iron oxide particles (SPIO). We examined the effects of long-term labeling, possible toxicological consequences and the possible influence of progressive concentrations of SPIO on chondrogenic differentiation capacity.

**Results:**

No influence of various SPIO concentrations was noted on human bone marow MSC viability or proliferation. We demonstrated long-term (4 weeks) *in vitro* retention of SPIO by human bone marrow MSCs seeded in collagenic sponges under TGF-β1 chondrogenic conditions, detectable by Magnetic Resonance Imaging (MRI) and histology. Chondrogenic differentiation was demonstrated by molecular and histological analysis of labeled and unlabeled cells. Chondrogenic gene expression (*COL2A2, ACAN, SOX9, COL10, COMP*) was significantly altered in a dose-dependent manner in labeled cells, as were GAG and type II collagen staining. As expected, SPIO induced a dramatic decrease of MRI T2 values of sponges at 7T and 3T, even at low concentrations.

**Conclusions:**

This study clearly demonstrates (1) long-term *in vitro* MSC traceability using SPIO and MRI and (2) a deleterious dose-dependence of SPIO on TGF-β1 driven chondrogenesis in collagen sponges. Low concentrations (12.5–25 µg Fe/mL) seem the best compromise to optimize both chondrogenesis and MRI labeling.

## Introduction

Because of their differentiation potentialities, mesenchymal stem cells (MSCs) provide a promising avenue to regenerate damaged tissues in articular diseases. One recent strategy is the use of MSCs for cartilage engineering [1], [2]. To assess the effects of MSC implantation, a non-invasive imaging technique was developed [3]. This allowed analysis of the bio-integration and bio-functionality of the engineered tissues and tracking of the labeled MSCs within the joint. MRI can measure the extent of a defect, the state of the surrounding tissue and the progressive repair longitudinally, at the tissue, cell and even the molecular level [4].

Several studies have addressed the topic of visualization and tracking of non-invasively transplanted cells by the use of MRI with superparamagnetic iron oxide (SPIO) particles as a contrast agent [5]–[8]. SPIO dramatically shorten the nuclear magnetic resonance T2 relaxation time [9]. Iron oxide labeled cells appear as hypo-intense areas in tissues on T2-weighted sequences [10], [11]. SPIO particles are introduced into the cells by endocytosis. A transfection agent (TA) such as poly-L-Lysine (PLL), which enhances cell adhesion to the surface of a culture dish during *in vitro* cell cultivation, could be used as a vehicle for iron particle transport into cells. PLL does not lead to cytotoxity as has been demonstrated by numerous studies [12], [13].

While the general consensus is that there is no effect of iron-oxide labeling on MSC morphological characteristics, viability, proliferation, or chondrogenic differentiation [5], [14], [15], the influence of SPIO-labeling on MSC chondrogenic differentiation has been more disputed in the last few years. It was shown that some experimental conditions could alter MSC-driven chondrogenesis. In fact, few reports have addressed simultaneously the dose-response cytotoxicity *in vitro* of iron labeling at various levels including cellular viability, chondrogenic differentiation at genic (PCR) and tissue levels (histology, MR signal at 3 T & 7 T). This is the object of the current multi-scale investigation.

## Materials and Methods

### Ethics statement, Isolation and culture of human bone mesenchymal stem cells (MSCs)

MSCs were isolated from femoral necks of patients undergoing total hip replacement (all for OA, all bone samples were received from our local bone bank, UTCT, see below) and were anonymous when researchers received them. This study was approved ethically and methodologically by our local Research Institution (Direction de la Recherche et de l'Innovation (DRCI) registration number UF 9757 - Contrat de Programme de Recherche Clinique (CPRC) - Cellules souches et chondrogénèse) and was conducted with informed patients (written consent, non-opposition) in accordance with the usual ethical legal regulations (decrees n 2007–1220 and AC-2008-49), in collaboration with our local bone bank (UTCT, Unité de Thérapie Cellulaire et Tissus, CHU Nancy, procedure validated by the French Drug Agency (ANSM)). All procedures were done in accordance with our authorization and registration number DC-2008-263 given by the National “Cellule de Bioétique”. The review board of DRCI and ANSM have approved ethically the informed consent.

### Labeling of MSCs in monolayers with superparamagnetic iron oxide particles (SPIO)

MSC labeling was initiated by the addition of medium that contained superparamagnetic iron oxide (SPIO) (Endorem, Guerbet S.A., Paris, France) and Poly-L-Lysine mixture (375 ng/mL, PLL Mw: 70,000–150,000, Sigma) and incubation was at 37°C/5% CO_2_ for 24 hours. Different concentrations of iron were tested for the labeling: without PLL, and PLL associated with 12.5 µg/mL, 25 µg/mL, 50 µg/mL, 100 µg/ml, 200 µg/ml, 400 µg/ml, 800 µg/ml and 1600 µg/ml. This choice of dose range was based on an analysis of the literature where most authors have used 25 to 50 µg/ml of iron.

### Lactate dehydrogenase (LDH) and caspase 3 activity

Cell viability was evaluated by the measurement of LDH activity at 24, 48 and 72 hours after SPIO labeling with the use of a Cytotoxicity Detection Kit (Roche, France) according to the manufacturer's recommendations. Apoptosis was evaluated by the measurement of caspase-3 activity in MSCs in the presence of SPIO labeling with or without PLL. Caspase-3 activity assay (Cell Signaling Technology) was used following the manufacturer's instructions. The caspase-3 Activity Kit is a fluorescent assay that detects the activity of caspase-3 in cell lysates. It contains a fluorogenic substrate (N-Acetyl-Asp-Glu-Val-Asp-7-amino-4-methylcoumarin or Ac DEVD-AMC) for caspase-3. During the assay, activated caspase-3 cleaves this substrate between DEVD and AMC, generating highly fluorescent AMC that can be detected using a fluorescence reader with excitation at 380 nm and emission at 440 nm. Cleavage of the substrate only occurs in lysates of apoptotic cells. MSCs were cultured in 96 well plates in the presence of the various concentration of Endorem with or without PLL for 24 h. Briefly, cells were harvested and re-suspended in 30 µl of cell lysis buffer. After lysis, 25 µl were mixed with 200 µL of assay buffer containing Ac-DEVD-AMC. After incubating at 37°C for 5 hours, RFU was ridden on a fluorescence plate reader with excitation at 380 nm and emission at 440 nm. Absorbance of each sample was determined by subtraction of the mean absorbance of the blank from the sample.

### Measurement of NO and PGE2 concentrations

The measurement of NO release was reflected by the determination of its stable end product, nitrite, in cell supernatants using the Griess reaction. Briefly, an aliquot (100 µl) of cell culture supernatant was incubated with 50 µl of 0.1% sulfanilamide in 5% phosphoric acid and 50 µl of 0.1% N-1-naphthylethylenediamine dihydrochloride. After 10 min incubation at room temperature, the absorbance was measured at 550 nm. ELISA was used to determine PGE2 concentrations in cell culture supernatants according to the manufacturer's protocol (Parameter, R&D systems, France).

### Prussian Blue staining

Cells on cover-slips were fixed in PFA 4% for 10 min, immersed in 2% potassium ferrocyanide and 2% HCl for 15 min, washed with distilled water, counterstained with kernechtrot (5 min), washed with distilled water and finally mounted in Pertex resin.

### Chondrogenic differentiation of SPIO-labeled MSCs in 3D culture

To determine whether iron labeling affected the chondrogenic differentiation potential of MSCs, 6 different concentrations of iron were tested: 0 (control), 12.5 µg/ml, 25 µg/ml, 50 µg/ml, 100 µg/ml and 200 µg/ml. To the best of our knowledge, concerning differentiation (and not toxicity), the maximal concentration used in the literature is 100 µg/ml. After incubation, MSCs were washed three times with PBS to remove any SPIO that had not been incorporated into the cells. Chondrogenic differentiation of human bone marrow MSCs was induced inside collagen sponge biomaterials from the “Symatese Biomatériaux” company, such sponges being commonly used in cartilage engineering [16], [17], and labeled for the clinics as medical device. Discs had dry dimensions of 5 mm diameter x 2 mm thickness and were derived from bovine hide. For each concentration of SPIO, cells were trypsinized and seeded in collagen sponges at a final concentration of 0.5×10^6^ cells/sponge. Collagen sponges were cultured for three days with chondrogenic medium supplemented with 10% FBS. Then, to induce chondrogenic differentiation, cells were cultivated in serum-free chondrogenic induction medium, that contained low-glucose DMEM supplemented with TGF-β1 at 10 ng/ml (Miltenni Biotec), L-ascorbic acid 2-phosphate (50 µg/ml, Sigma), sodium pyruvate (100 mM Invitrogen), proline (40 µg/ml, Sigma), dexamethasone (10^−7^ M), penicillin-streptomycin and 1% ITS^+^ Premix (Beckton Dickinson). Cells were cultured in this chondrogenic differentiation media for 28 days (D28). Each TGF-β1 condition (SPIO-labeled and unlabeled sponges) was compared to sponges cultured without TGF-β1 (ITS condition).

### Mitochondrial activity

Mitochondrial activity in collagen sponges was evaluated by an MTT (3(4,5-Dimethylthiazol-2-yl)-2,5-diphenyltetrazolium bromide) assay at D28. 400 µl of culture medium and 100 µl of MTT solution (5 mg/ml MTT in PBS) were added per collagen sponge and plates were incubated in the dark at 37°C under 5% CO_2_ for 4 hours. The intense purple coloured formazan derivative formed during active cell metabolism, was eluted and dissolved in a solution containing sodium dodecyl sulfate with dimethylformamide. The absorbance was measured at 580 nm. Mitochondrial activity results were normalized by DNA quantisation in each sponge. To evaluate total DNA quantity, Hoechst 33258 dye (Molecular Probes) was used. Working assay solution was prepared fresh prior to each assay by mixing 1 µl of concentrated dye stock solution (0,1 mg/ml) for every 1 ml of assay buffer required resulted in a final Hoechst 33258 concentration of 0,1 µg/ml. Cells were lysed by freeze-thawing in 100 µl of a buffer containing 10 mM Tris, 1 mM EDTA and 0,1 M NaCl, pH 7,4. Then, 2 ml of working Hoechst solution were added in each sample. Results were extrapolated from a standard curve based on calf thymus DNA (D-3664, Sigma, France) ranging from 0 to 0.5 µg/ml. Samples were measured fluorometrically (F2000 Fluorescence Spectrophotometer, Hitachi) at excitation wavelength of 348 nm and emission wavelength of 456 nm.

### Gene expression analysis

On D28, unlabeled and labeled cells were collected from collagen sponge constructs (TGF-β1 and ITS conditions) by collagenase enzymatic digestion and centrifuguing. Chondrocyte differentiation was assessed by mRNA expression levels of different collagens (total Coll II, Coll IIb isoform as the more chondral specific procollagen resulting from alternative slicing of exon 2 [18]), and Coll X, aggrecan, COMP, Sox9 and osteogenic markers (alkaline phosphatase, osteocalcin), and was compared to ITS on D28.

The total cell RNA was extracted using the RNeasy mini kit (QIAGEN, France), according to the manufacturer's instructions as previously described [19]. The RNA was quantified spectrophotometrically and reverse transcribed with the iScript cDNA Synthesis Kit (Bio-Rad, France) and samples were placed following recommendations. After cDNA synthesis, quantitative RT-PCR was performed with a Lightcycler (Roche). cDNA of each gene was obtained according to the manufacturer's instructions (Quiagen, purification kit), dosed, then diluted to obtain a standard curve of the 10^−3^ to 10^−6^ µg/ml range, which permits quantification of the expression of the gene of interest. For each condition, the signal of the RP29 housekeeping gene was determined once for each cDNA sample, and this was used to normalize the findings for all other genes.

### MR exploration of 3D constructs

For MR exploration, two different sizes of sponges were used, depending on the resolution obtained at 3T and 7T: Sponges with a diameter of 5 mm and a thickness of 2 mm were used at 7T and sponges with a diameter of 1 cm and a thickness of 4 mm at 3T.

#### 7T MRI

On D28, TGF-β1 and ITS sponges labeled or not with SPIO (0–12.5–25–50–100 and 200 µg Fe/mL) underwent an MR exploration to determine T2 values of synthesized matrix at 7T (Biospec 7T Imager, Brucker Biospin MRI GmbH, Etlingen, Germany) with a maximum gradient strength of 0.4 T/m. A quadrature volumic 32 mm RAPID coil (Biomedical GmbH, Rimpar, Germany) was used for acquisitions. MRI experiments were conducted with the following acquisition parameters: multi-slice multi-echo T2 mapping sequences (180° flip angle), TR/TE: 2500/10 ms, 16 echoes (from 10 to 160 ms), FOV: 3 cm, slice thickness: 1 mm, NEX: 4, and acquisition time: 32 min. The MR images were obtained using a matrix size of 256×256 with a resolution of 0.0117 cm/pixel. Regions of interest of the appropriate size were chosen within the samples and ParaVision 5.1 software (Bruker BioSpin MRI GmbH, Ettlingen, Germany) was used to determine T2 values on functionalized biomaterial MR images. T2-values were obtained from the multiple spin-echo sequence by fitting a mono-exponential decay function to the 16 data points (echoes). T2 was calculated according to the equation S = S_0_e^−TEi/T2^, where S stands for the mean signal intensity at echo time TE of echo i, and S_0_ is the signal intensity at the origin, just after the first 90° radio-frequency pulse.

#### 3T MRI

On D28, TGF-β1 and ITS SPIO-labeled or not labeled sponges (0–12.5–25–50–100 and 200 µg Fe/mL) were placed in 24 well plates. MR Imaging of MSC-seeded sponges was performed on a clinical 3T MRI system (General Electric) using multi-slice multi-echo 2D T2 mapping sequences. MRI experiments were conducted with the following acquisition parameters: multi-slice multi-echo T2 mapping sequences (180° flip angle), TR/TE: 1500 ms, 8 echoes (from 10 to 160 ms), slice thickness:1 mm, NEX: 3 and acquisition time: 32 min. The MR images were obtained using a matrix size of 288×224 reconstructed by interpolation in 512×512 with a resolution of 0.234 mm (pixel spacing). Regions of interest of the appropriate size were chosen within the samples. Determination of T2 values was obtained by the reconstruction of T2 map from 8 echoes sequences by using of FuncTool Software (GE Medical systems, Europe) and especially the Cartigram software package.

### Histology

On D28, collagen sponges were fixed with PFA 4% during 24 hours at 4°C, dehydrated, embedded in paraffin, and cut into 5-µm thick sections with a Leica micro-tome (RM2135, Leica, France). Each specimen was stained with Haematoxylin-Erythrosin-Saffron for morphology, Alcian Blue for proteoglycan content and Sirius red for collagen content and was visualized on an optical microscope (DMD 108, Leica, France). The Prussian Blue staining technique was used for SPIO detection as described above.

### Immuno-histochemistry

Immuno-histochemistry was performed according to LSAB+ kit (HRP, Dako) based on avidin-biotin techniques, with antibodies for type II collagen to assess the degree of chondrogenic MSC differentiation. Primary monoclonal antibodies (collagen 1 (interchim) and 2 (Labvision)) were used. Paraffin-embedded tissue sections of 5 µm were de-paraffinized, treated with pepsin (0.4% w/v, Sigma) for 30 min at room temperature and incubated with a hydrogen peroxide block solution for 5 min to block endogenous peroxidase, according to the manufacturer's recommendations. Finally, the sections were counter-stained with Prussian Blue for 15 min and with Hematoxylin for 30 sec and mounted with Eukitt resin.

### Data analysis

Values are expressed as mean ± SD. Each experiment was carried out at least twice in triplicate. Cytotoxicity, caspase 3 activity and relative gene fold expressions were analyzed by a one-way ANOVA test followed by the Bonferroni's post hoc test. Mitochondrial activity and T2 values of TGF-β1 and ITS SPIO-labeled sponges were analyzed by a 2-way ANOVA test followed by the Bonferroni's post hoc test (concentration vs culture medium, namely ITS and TGFβ1). A p-value of <0.05 was considered to indicate a significant statistical difference.

## Results

### Cellular viability, NO and PGE2 production of SPIO-labeled MSCs in monolayers

**Figure 1:**
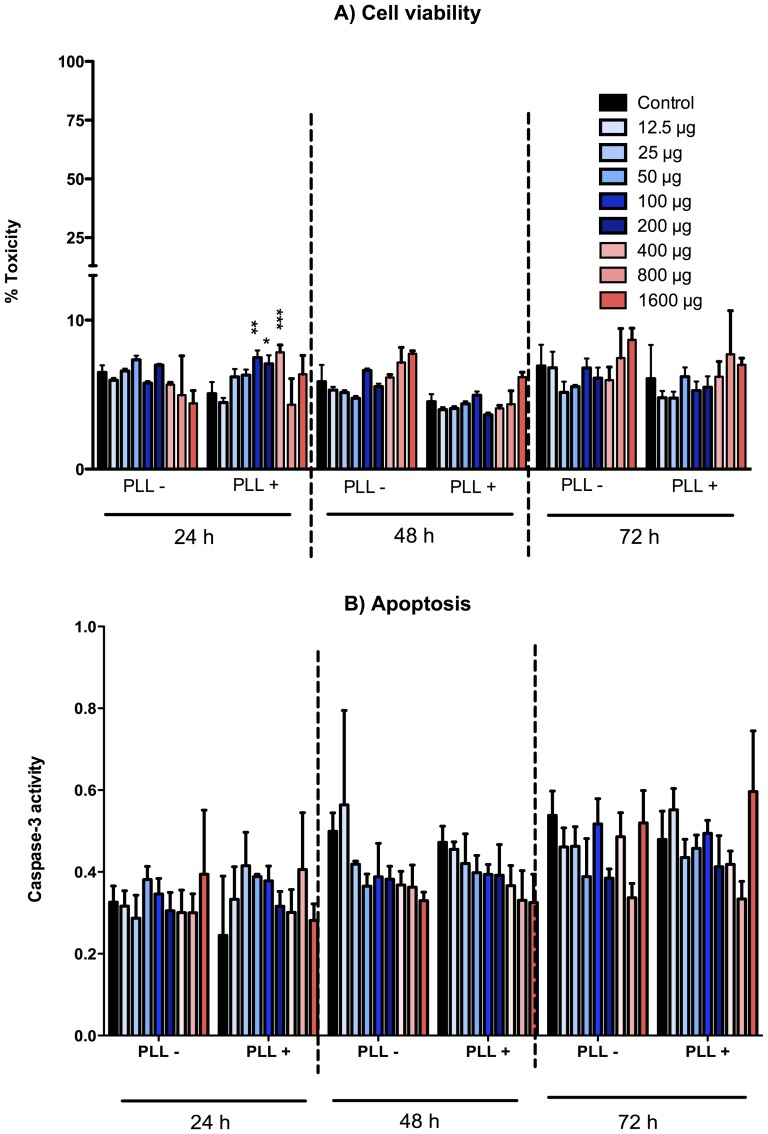
Viability and apoptosis of SPIO labeled human bone MSC (2D Culture). A) Lactate dehydrogenase viability assay. B) Caspase 3 activity. Cell death and apoptosis were minimal in MSC cultured in media only (control) under different concentration of superparamagnetic iron oxide particles (SPIO from 12.5 to1600 µg ƒe/mL). All comparisons of labeled conditions (SPIO) were performed versus the control condition (0) with a 1-way ANOVA followed by a Bonferonni's test. Data are mean ± sd. *p<0.05, **p<0.01, ***p<0.001. The experiment was carried out twice in triplicate.

Progressive SPIO concentration did not enhance LDH activity versus controls at 48 and 72 h. A slight cyto-toxicity was observed at 24 h for some high SPIO concentrations PLL+, but not for the highest (800 & 1600 µg Fe/ml). In all conditions, less than 10% toxicity was observed, even for the highest SPIO concentrations tested (Figure 1A). In similar conditions, no apotosis was detected, when assessed with with caspase 3 activity, versus their own controls (Figure 1B). After 24 hours of exposure to SPIO, NO production significantly increased to the highest concentrations of 400 to 1600 µg Fe/ml with or without PLL (9 to 12 fold without PLL and about 4 to 7 fold with PLL higher compared to 0 µg Fe/ml, p<0.05). At 48 hours, we noticed an increase of NO production of 60%, with or without PLL, only for the highest concentration of SPIO tested (1600 µg Fe/ml, p<0.05). NO production did not vary later than 48 hours after treatment with SPIO. However, NO concentration was low, below 10 µM (data not shown). This indicates that no significant cytotoxicity was detected in all concentrations of SPIO tested. In addition, SPIO labeling did not stimulate basal PGE2 concentration in any batch studied (data not shown).

### SPIO internalization into MSCs in monolayers

**Figure 2:**
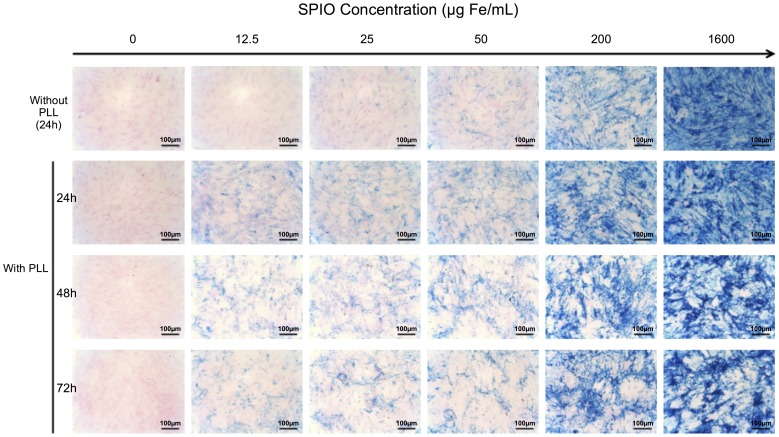
Histological visualization of human bone MSC (2D culture) using Prussian Blue staining. Staining was performed at 24(SPIO). Staining increases with SPIO concentration. It should be noted that the SPIO uptake by MSCs is amplified by the use of the Poly-L-Lysine (PLL) transfection agent.

Prussian Blue staining provided the information that even without PLL the iron particles were internalized into cells. But the association of SPIO labeling with PLL greatly improved this internalization as depicted by stain intensity gradation. The internalization increased with rising SPIO concentration and iron remained in the cells until at least 72 hours post-exposure (Figure 2).

### TGF-β1 driven differentiation of SPIO-labeled MSCs

**Figure 3:**
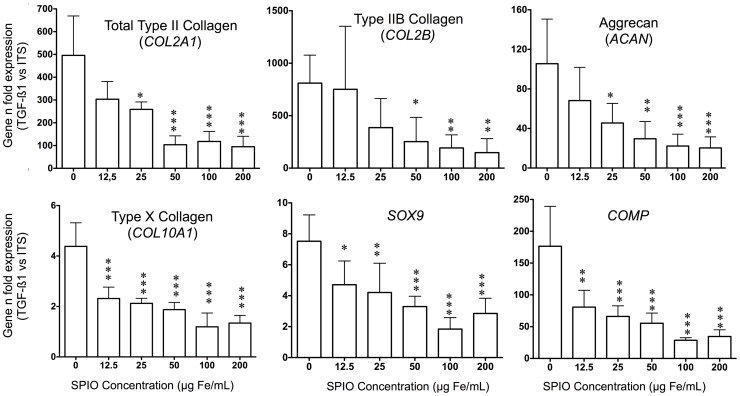
Normalized gene expression (TGF-β1 versus ITS) at D28 with various SPIO concentrations. All comparisons of labeled conditions (SPIO) were performed versus the control condition (0) with a 1-way ANOVA followed by a Bonferonni's test. Data are mean ± sd. *p<0.05, **p<0.01, ***p<0.001. The experiment was carried out twice in triplicate.

As predicted, without SPIO TGF-β1 induced a typical chondrogenic differentiation of MSCs seeded 3D in collagen sponges (Figure 3): we observed a highly significant over-expression of global type II collagen (495 fold versus ITS condition, p<0.001) and especially for type IIB collagen, which is typically expressed in differentiating and adult cartilage (810 fold, p<0.001). Increases were also found for type X collagen (4.4 fold, p<0.001), aggrecan (105.6 fold, p<0.001), Sox 9 (7.52 fold, p<0.001) and COMP (176.5 fold, p<0.001). In contrast, in our experimental conditions (chondrogenic medium), TGF-β1 supplementation did not significantly induce the over-expression of osteogenic markers such as alkaline phosphatase or osteocalcin (NS, data not shown).

Figure 3 also demonstrates the dose-dependent repression of progressive SPIO labeling on these 6 typical chondrogenic genes, beginning at the lowest concentration for SOX9, COMP and type X Collagen) statistically significant at 25–50 µg Fe/mL. All TGF-β1 sponges exerted significant differences compared to ITS sponges for each condition tested.

### Mitochondrial activity in collagen sponges

**Figure 4:**
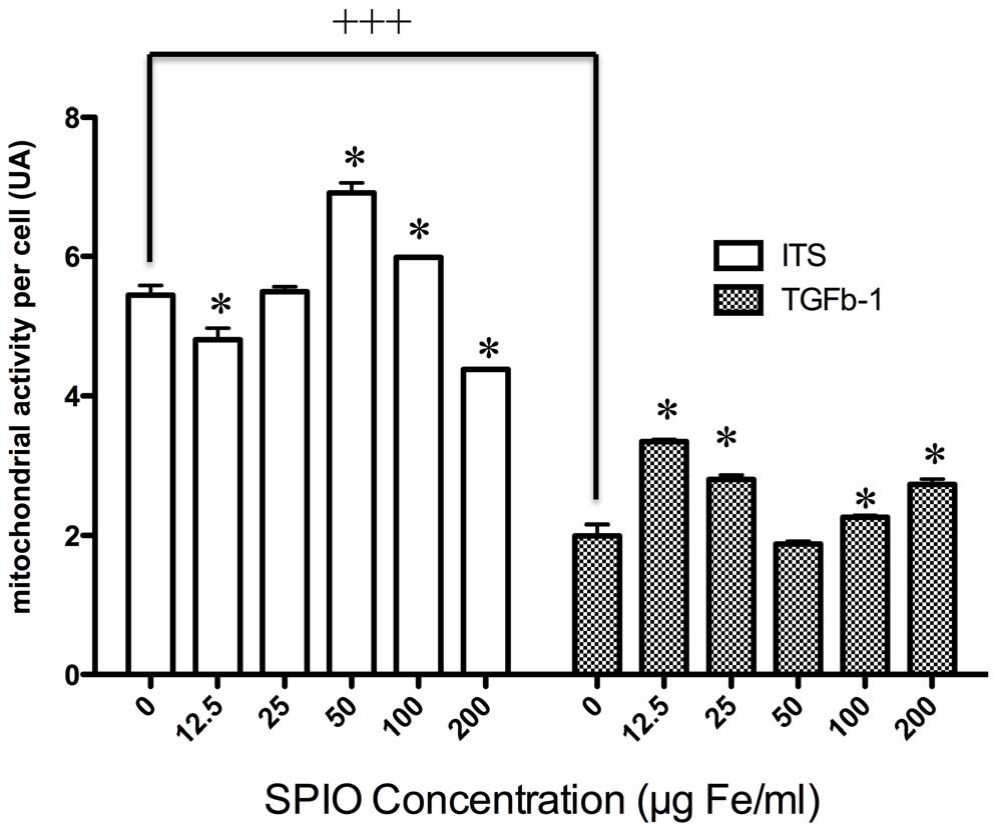
Normalized mitochondrial activity of sponges seeded with undifferentiated and differentiated MSCs performed at D28 using MTT assay normalized with DNA content of each sponge. All comparisons of labeled conditions (SPIO) were performed versus the control condition (0) with a 2-ways ANOVA (SPIO concentration and medium, ITS vs TGFβ) followed by a Bonferonni's test. Data are mean ± sd. *p<0.05. The experiment was carried out four times. There is a significant interaction between the 2 factors (SPIO concentration and culture medium, ITS and TGFβ-1 conditions)

In control sponges, without labeling, TGF-β1 induced a significant decrease of normalized mitochondrial activity compared to ITS, inherent to the chondrogenic differentiation that predominates over proliferation (p<0.01). Upon SPIO labeling, although significant inter-group variations, no significant deleterious dose-response on normalized mitochondrial activity was detected in either ITS or TGF-β1 conditions (Figure 4).

### Histological analysis of collagen sponges

**Figure 5:**
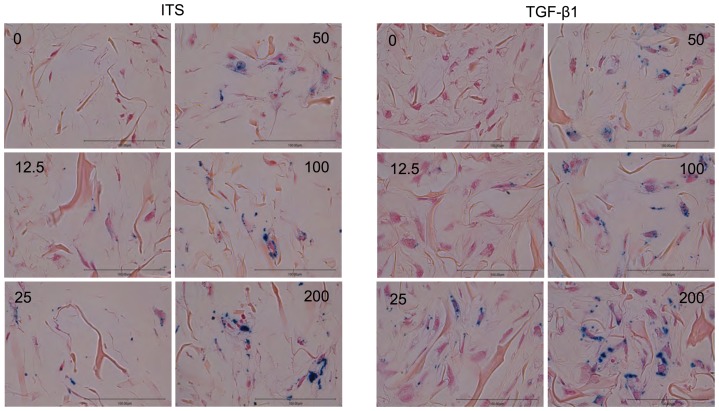
Histological analysis of sponges seeded with undifferentiated and differentiated MSCs performed at D28 using Prussian Blue staining. When compared to control (ITS 1%), TGF-β1 induced a greater and more homogeneous synthesis of the extracellular matrix. Non labeled cells do not contain any iron. Dose-dependent intracellular Prussian blue staining was observed in collagen sponge colonized by mesenchymal stem cells at 28 days after SPIO labeled different concentrations of iron: 0, 12.5, 25, 50, 100 and 200 µg Fe/ml. Bar scale corresponds to 100 µm.

To visualize the internalization of iron particles after the cells had spent 28 days in collagen sponges, Prussian blue staining was performed (Figure 5). In unlabeled control sponges (TGF-β1 and ITS), no positive staining was observed within the cells. In contrast, after labeling, iron particles were retained intra-cellularly at this time. No extra-cellular staining was depicted. The accumulation of iron particles appeared to be visually proportional to the SPIO concentration used, in both ITS and TGF-β1 conditions: at the highest concentration, about 95% of MSCs were labeled intra-cellularly in both conditions. The presence of PLL did not impair chondrogenic differentiation of MSCs and ancillary chondral extracellular matrix synthesis was well distributed throughout the sponges.

**Figure 6:**
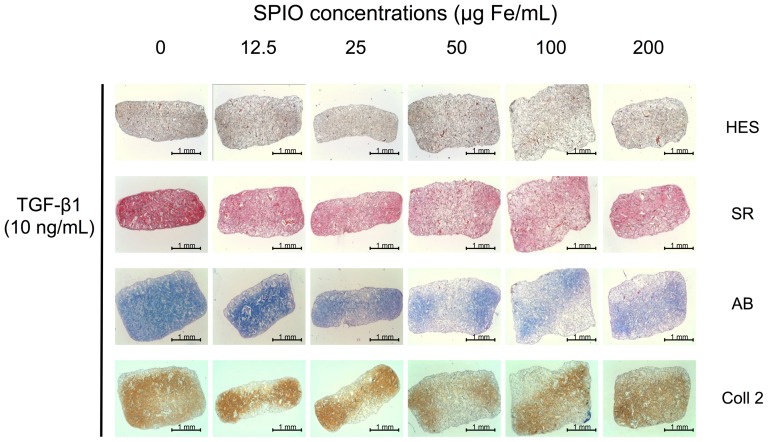
Histological and immuno-histochemical analysis of sponges seeded with differentiated MSCs performed at D28. When compared to control (0) SPIO addition led to proteoglycan depletion beginning at 25 µg Fe/mL (Acian Blue, AB), which increased as the SPIO concentration was raised. Similarly, SPIO labeling exerted a deleterious effect on collagen synthesis, as illustrated by Sirius Red (SR) staining, most specifically on collagen II synthesis. No effect is depicted on cell viability assessed with Hematoxylin-Erythrosin-Saffron

Alcian Blue staining was performed at D28 to assess the proteoglycan content of the sponge-associated cells (Figure 6). In unlabeled control TGF-β1 sponges, GAG was abundant and homogeneous. After labeling, blue stain attenuation began at 25 µg Fe/mL, was more marked at 50 µgFe/mL and then remained stable.

Sirius Red staining that gives information about fibrillar collagen content was also carried out on D28 (Figure 6). In TGF-β1 controls, MSCs synthesized a dense collagenic extra cellular matrix (ECM). SPIO labeling beginning at 50 µg/ml led to a decrease of ECM staining. Immuno-staining of type II collagen, which is more specific, also confirmed a staining loss slightly beginning at 12.5 µg/ml and more pronounced at 50 µg/ml.

### In vitro MRI tracking of labeled cells seeded into collagen sponges

**Figure 7:**
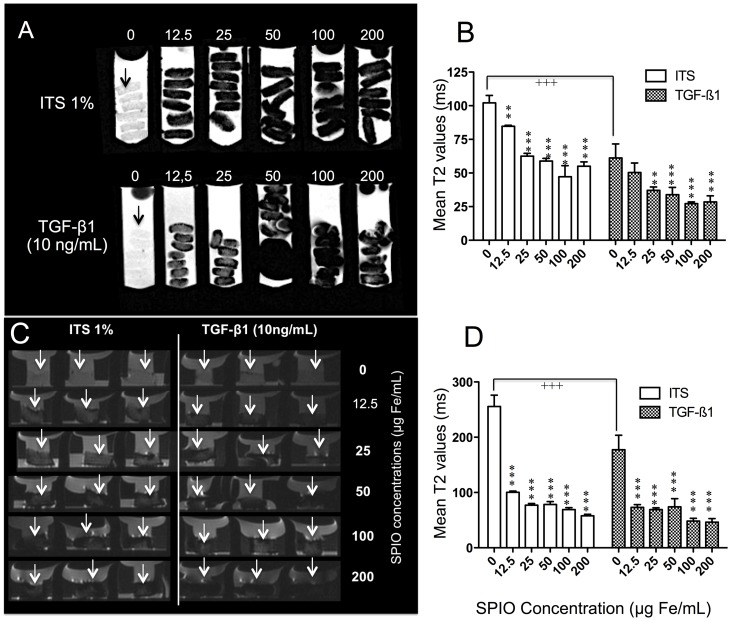
MR assessments of collagen sponges at 7 (A,B) and 3T (C, D). A. 7T MR images of sponges (black arrow) seeded with undifferentiated (ITS 1%) or differentiated (TGFβ-1 (10 ng/mL)) human bone MSCs labeled with increasing SPIO concentration, after 28 days *in vitro*. SPIO are homogeneously distributed in the sponges and the T2 signal decreases as the SPIO concentration increases for both conditions. B. Representation of mean relaxation time values of functionalized biomaterials on D28 *in vitro* at 7 T. Both ITS (0) and TGFβ-1 (0) are significantly different (+++, p<0.001, Student's t test). For each condition, T2 values of labeled sponges were compared to their own control (ITS 0 and TGF respectively) with a 2 way ANOVA followed by a Bonferonni's test. Data are mean ± sd. *p<0.05, **p<0.01, ***p<0.001. The experiment was done twice in triplicate. There is a significant interaction between culture medium and SPIO concentrations. C. 3T MR images of sponges (white arrow) seeded with undifferentiated (ITS 1%) or differentiated (TGFβ-1 (10 ng/mL)) human bone MSCs labeled with increasing SPIO concentrations, after 28 days *in vitro*. D. Corresponding T2 values. ITS (0) and TGFβ-1 (0) are significantly different (+++, p<0.001, Student's t test). For each condition T2 values of labeled sponges were compared with their own control (ITS 0 and TGF respectively with a 2 way ANOVA followed by a Bonferonni's test. Data are mean ± sd. *p<0.05, **p<0.01, ***p<0.001. The experiment was carried out twice in triplicate. There is a significant interaction between culture medium and SPIO concentrations.

MR scans of labeled MSC-seeded sponges were performed (D28) on 7T (experimental) and 3T (clinical) imagers to assess their respective MR signal inherent to the progressive iron uptake (Figure 7). As expected, a progressive visual MR signal loss was characterised by a gradual darkness depending on SPIO concentration at both 3 and 7 T. Black regions in the scaffolds without labeled MSCs were attributed to air bubbles.

#### 7T MR

In unlabeled control groups, the mean T2 value of TGF-β1 sponges (Figure 5B) was significantly lower (40%) than the T2 of ITS sponges. In labeled groups, the distribution of iron particles was homogeneous and, as expected, the MR signal decreased as the SPIO concentration increased, for both ITS and TGF-β1 conditions (Figure 5 A–B). The visual observations were confirmed by T2 values. For the ITS sponges, in which MSCs remained undifferentiated, the mean T2 value was significantly reduced by 15.3% at the lowest concentration of 12.5 µg Fe/mL (T2  =  102.10 ms) (p<0.01) (Figure 5B). Concerning TGF-β1 sponges, a significant reduction (39.3%; p<0.01) began at the concentration of 12.5 µg Fe/mL (T2  =  61.23 ms), decreasing at 25 µg Fe/mL and then remaining stable.

#### 3T MR

We observed visually on MR scans an inhomogeneity of the SPIO repartition into the sponges, SPIO being preferentially located at the periphery (Figure 5C). In control cells, TGF-β1 sponges exerted a significantly lower T2 (177.34 ms; p<0.001) than ITS sponges (T2: 255.64 ms) (Figure 5D). After labeling, in both ITS and TGF-β1 sponges, there was a strong significant inhibition of T2 values beginning at 12.5 µg Fe/mL (60.7% (p<0.001) and −58.7% (p<0.001)) respectively, and then remaining stable.

## Discussion

Our study has assessed the dose-effect of a labeling of human mesenchymal stem cells by a range of growing SPIO concentrations. Effects on biological functions, chondrogenic differentiation and labeling MR performance were measured. To assess the influence of SPIO labeling on chondrogenic differentiation, MSCs were cultured in collagen sponges (3D) in the presence of TGF-β1 for 28 days versus ITS conditions. The MR ability to detect labeled cells in collagen sponges was performed on D28 by using 3T and 7T MRI.

In a preliminary step, we investigated human bone marrow MSC behaviour in monolayer culture when MSCs were exposed to a dose of SPIO ranging from 0 to 1600 µg Fe/ml. Neither cytotoxicity, nor cell death or apoptosis were detected under these conditions, even at the highest concentration of 1600 µg Fe/ml tested, and there was no significant concomitant production of NO and PGE2. Similarly, in a previous study, Arbab [5] demonstrated that a lower concentration-range (1 to 100 µg Fe/ml) did not affect viability or toxicity of various cell lineages including human MSCs. Furthermore, an SPIO exposure from 2 to 168.75 µg Fe/ml did not affect chondrocyte viability [14]. More recently, a dose range of 2.5 to 25 µg ferumoxide/cm^2^ was shown to have no negative effect on human marrow bone MSC viability [15]. Another team investigated the effect of a concentration range of 1 to 100 µg Fe/ml of SPIO direct labeling on immortalized human MSCs and proved that the labeled cells did not go through apoptosis or produce elevated levels of ROS or MMP [20]. Other works have pointed out this lack of SPIO toxicity on murine or human MSCs, with concentration ranges from 0 to 1000 or even 1600 µg Fe/mL [21], [22]. A similar study [23] showed that, when compared with unlabeled controls, labeled MSCs exhibited an unaltered viability and proliferated similarly. All these results are concordant with the conclusion of a lack of SPIO direct toxicity in this concentration range, in terms of necrosis or apoptosis. This is reassuring as SPIO are accepted by drug agencies for intravenous injections.

Additionally, the interest of PLL as a transfection agent to facilitate cell labeling has been confirmed [24]. PLL increased the efficiency of labeling without significant alteration of the biological functions of MSCs. Significantly increased cell labeling by SPIO was demonstrated by augmented Prussian Blue staining as previously demonstrated [5]. We also showed that MSCs could internalize large amounts of iron particles without saturation in a dose-dependent manner over the range used here. Prussian Blue staining was progressively diluted over time, probably due to cell proliferation. The temporal profile of this decrease correlated to the dose and to the incubation time as previously mentioned [25]. We showed herein that without SPIO the mitochondrial activity in the TGF-β1 condition was strongly reduced on D28 versus undifferentiated MSC (ITS), demonstrating that TGF-β1 promotes cellular differentiation over proliferation. SPIO labeling did not exert any deleterious dose–dependent effect on cell viability or mitochondrial activity.

The study of cartilage gene expression corroborates the fact that TGF-β1 versus ITS induces typical MSC-driven chondrogenesis [26] in collagen sponges (on D28) as reflected by an increased expression of type II collagen (especially COL2 IIB), Sox 9, COMP and aggrecan mRNAs. Type X collagen gene over-expression, reflecting hypertrophy, is a classical phenomenon during 3D MSC-driven chondrogenesis [19], depending on pre-conditionning [27], culture medium, TGFβ isoform [28], oxygen tension, co-culture, epigenetics and biomaterial composition [29]. Control of hypertrophic differentiation is an actual challenge for cartilage engineering where stable phenotype is desired. On the other hand, markers such as osteocalcin and alkaline phosphatase mRNAs were expressed at very low rates suggesting that MSCs are not engaged in an osteogenic pathway. Additionally, we observed a concentration-dependent inhibition of these cartilage-specific gene expressions concomitant to progressive SPIO labeling: type 2 collagen, aggrecan. inhibited at 25–50 µg Fe/mL. Type X Collagen, Sox 9 and COMP gene expressions were strongly inhibited at all concentrations. With this in mind, a low concentration of SPIO (12.5 µg Fe/mL) seems suitable for *in vivo* tracking of MSC-driven chondrogenesis. To translate these results obtained at the gene level to tissue organization, we performed histological and immuno-histochemical analysis of chondrogenic constructs under progressive SPIO exposure. Using Prussian Blue staining, we showed, as observed in 3D conditions that iron particles were perfectly and homogeneously distributed in MSCs on D28 in a concentration-dependent manner in MSC-seeded sponges under TGF-β1 conditions. Furthermore, collagenic staining with Sirius Red, as well as immuno-staining of type II collagen, was altered at concentrations above 25–50 µg Fe/mL. Additionally, Alcian Blue staining that reflects GAG content was altered above a 25 µg Fe/mL concentration.

To date, only a few studies have investigated the effect of SPIO on MSC chondrogenic differentiation, and these showed conflicting results. Kostura [23] observed that 25 µg Fe/ml of SPIO led to a partial inhibition of chondrogenesis in pellets of human bone marrow MSCs at the tissue level. However, only one iron concentration and simply histological assessment were used. Bulte [30], in a short letter, suggested that this toxicity is due to iron itself and not to the transfection agent and may be dose-dependent, as in our study. Discordant results were described by Heymer [31], working in collagen hydrogels at 1.5 mM, who reported no SPIO influence on chondrogenic gene expression, but a slightly more intense staining intensity in the pellets of unlabeled cells. In this way, Boddington [32] did not observe any influence of 100 µg Fe/mL on MSC-induced chondrogenesis (pellet) as assessed histologically, but found a significantly reduced GAG production in labeled cells. Other contradictory studies did not demonstrate any deleterious influence on MSC differentiation. Arbab [33] did not observe any cyto-toxicity or chondrogenesis inhibition at a concentration of 100 µg/ml SPIO. Jing et al [4], in an interesting *in vitro* and *in vivo* study tested a concentration of 50 µg Fe/ml and did not notice any effect on the chondrogenic differentiation of rabbit MSCs. Similarly Farrel [14], when using 6.25 µg Fe/ml on human MSCs and multi-lineage differentiations (including chondrogenic) found no effect. Yang et al. [20] did not show any influence of 0–100 µg Fe/ml on TGF-β3 driven chondrogenicity of immortalized human MSCs. Also, Van Buul [15] did not observe any influence of SPIO on GAG production and type II collagen gene expression.

In clinics, the impact of local or systemic iron overload on chondrocytes is well known (haemophiliac arthropathy, haemochromatosis) but is poorly understood. Since the pioneering work of Farrel [34], which claimed that iron labeling (1–168.75 µg) has no effect on de-differentiated (P2) and re-differentiated chondrocytes at the metabolic, genic and proteic levels, more recent results are contradictory. Foldager [35], working on freshly harvested human chondrocytes in alginate beads reported a negative effect of cell labeling with SPIO particles on SOX9 and Coll 2 gene expression, but this was without influence on histological findings. Since this report, Saha [36] explored the SPIO influence on both MSCs and chondrocytes (neo-natal and adult). They observed a temporary down-regulation, but at the end of the experiment ACAN, SOX9 and COL2A1 expressions were similar in labeled and unlabeled cells (three cell lines tested; one concentration 0.5 µM FemL), suggesting a transient and target cell-dependent down-regulation. Ramaswamy [37] reported no influence of chondrocyte labeling on ECM production in hydrogel. These discrepancies may depend on the SPIO ± transfection agent used, its concentration, the 3D culture used, the endpoint, the scaffold, the growth factor, or the parameter studied.

T2 relaxation time is the transverse relaxation time that is dependent on the interaction between spins and tissue hydration [9]. In addition, in cartilage engineering T2 appears to be sensitive to the structural characteristics of the repair tissue, as reflected in our study *in vitro* by the significant difference of T2 in ITS versus TGF-β1 unlabeled chondrogenic sponges at both 7T (experimental) and 3T (clinical) scans. As, in ITS condition, there is less extra-cellular matrix production, it remains more water, and subsequently the T2 value increases. Furthermore, SPIO labeling diminishes T2 values at 7T in a dose-dependent manner, but more strongly at 3T, where there is saturation from the lowest SPIO concentration tested. Such a dose-dependent response has already been reported at 3T in labeled porcine chondrocytes [38]. Interestingly, our results concerning the reduction of T2 relaxation time at D28, as previously observed for MSCs [39] or chondrocytes [37], are encouraging in the perspective of a post-operative follow-up in demonstrating that SPIO remain intra-cellular during this period, as demonstrated histologically. As observed on day 28 in collagen sponges, there is a dose-dependant intracellular intracytoplasmic distribution of the fixed initial iron to the progeny of the proliferating cells without release to the surrounding media. With this in mind since on T2 weighted sequences normal cartilage appears as grey, a chondrogenic labeled implant will be easily detectible as a dark spot in the repair area *in vivo*, as previously observed subcutaneously [14] or intra-articularly in mice [40], rabbit [4] or pig knees [15], [38]. Most of these *in vivo* studies were performed by using a range of SPIO concentrations of 10 to 50 µg/ml with a MR follow-up varying from 1 to 12 weeks.

### Limitations of the study

We recognize several limitations of our study. Firstly, we examined MSC differentiation *in vitro*; differentiation processes are more complex *in vivo* and have to be reevaluated in this context. Secondly, we focused our study on the contrast agent Endorem, and further studies have to investigate the effect of other MR contrast agents on MSC differentiation. Thirdly, we examined human bone MSCs and other MSC types may be more or less sensitive to contrast agent labeling. Lastly, we considered a SPIO dose-ranging based on an analysis of the literature, as most of the published studies are performed at 25–50 µg Fe/ml. We observed no significant influence of our lowest concentration (12.5 µg/ml) on typical chondrogenic genes, namely type II collagen and agrecan, but other parameters like type X collagen, COMP gene expressions are affected by all concentrations.

***In conclusion***, this multi-scale *in vitro* study clearly demonstrates (i) long-term *in vitro* MSC traceability using SPIO and MRI and (ii) a deleterious dose-dependence of SPIO on TGF-β1 driven chondrogenesis in collagen sponges not linked to any cytotoxicity or proliferation problem. Low concentrations (12.5–25 µg Fe/mL) seem the best compromise to optimize both chondrogenesis and MR labeling for post-surgical follow-up.
